# Meta-analysis of repetitive transcranial magnetic stimulation combined with task-oriented training on upper limb function in stroke patients with hemiplegia

**DOI:** 10.1097/MD.0000000000033771

**Published:** 2023-06-02

**Authors:** Xiaoming Xi, Hujun Wang, Liang Han, Mengmeng Ding, Jinglu Li, Chenye Qiao, Linlin Zhang, Zongjian Liu, Shuyan Qie

**Affiliations:** a Rehabilitation Center, Beijing Rehabilitation Hospital Affiliated to Capital Medical University, Jinan, Shandong Province, China; b School of Rehabilitation Medicine, Shandong University of Traditional Chinese Medicine, Jinan City, Shandong Province, China; c School of Beijing Rehabilitation Medicine, Capital Medical University, Beijing, Fengtai District, China.

**Keywords:** Meta-analysis, repetitive transcranial magnetic stimulation, stroke, task-oriented training, upper limb function

## Abstract

To evaluate the effect of repetitive transcranial magnetic stimulation (rTMS) combined with task-oriented training (TOT) on upper limb function in stroke patients with hemiplegia.

**Methods:**

A systematic review and meta-analysis was performed using PRISMA guidelines. Computer searches of PubMed, Cochrane Library, Embase, Web of science, China Knowledge Network, Wanfang, and Wipu databases were conducted from the time of database creation to October 27, 2022. Clinical trials meeting the inclusion criteria were screened, with rTMS combined with TOT in the test group and other therapies in the control group. Literature screening and data extraction were performed independently by 2 investigators, and meta-analysis was performed using Stata software after quality evaluation of the literature.

**Results:**

Meta-analysis results showed that repeated transcranial magnetic stimulation combined with TOT was more effective in box and block test (*I*2 = 0%, *P* = .820, 95% confidence interval [CI] [−0.20, 0.88]), Fugl-Meyer Assessment (*I*^2^ = 0%, *P* = .569, 95% CI [0.88, 1.26]), and modified Barthel Index (*I*^2^ = 39.9%, *P* = .189, 95% CI [0.45, 1.03]) were not significantly different from controls, and the efficacy was significantly better in motor evoked potentials (*I*^2^ = 86.5%, *P* < .001, 95% CI [−1.38, −0.83]).

**Conclusions:**

Data analysis clarified the efficacy of rTMS) combined with TOT on upper extremity motor function disorders after stroke, but there was no significant difference between the efficacy in box and block test, Fugl-Meyer Assessment, and modified Barthel Index and the efficacy in motor evoked potentials between rTMS and the control group, suggesting that the neuro plasticizing effect of rTMS may translate into functional improvement by promoting neuro electrical signaling.

## 1. Introduction

Stroke is the second leading cause of death and the third leading cause of disability in the world population, costing an estimated more than $721 billion, or 0.66% of the global gross product,^[1]^ and causing a huge economic loss. Available literature suggests that 30-66% of stroke survivors have persistent upper limb injuries.^[2]^ Modern exercise therapies can accelerate upper extremity motor control and functional improvement in stroke patients, but still do not lead to full functional recovery in patients with moderate to severe strokes.^[3]^ The most challenging issue in stroke rehabilitation is the recovery of arm and hand function, with only 20% of patients recovering normal hand function and <50% using the affected arm and hand in any activity.^[4]^ Considering the importance of upper limb function for activities of daily living, it is necessary to investigate novel therapies to improve upper limb function. Repetitive transcranial magnetic stimulation (rTMS) is a noninvasive brain stimulation method that has incredible therapeutic potential as objective clinical and basic scientific data show that it triggers neuronal plasticity and enhances synaptic transmission.^[5]^ rTMS is divided into low-frequency and high-frequency stimulation, with low-frequency stimulation suppressing excitability on the non-affected side and high-frequency stimulation can promote excitability on the affected side.^[6]^ Currently, rTMS has been widely used in stroke rehabilitation, with good efficacy in motor function, cognitive ability, sleep quality, dysphagia, and depression. Task-oriented training (TOT) has been reported to achieve significantly good results in motor control and task-specific performance related to daily activities.^[7]^ the main limitation of TOT is that the task is repeated in an appropriate manner and most patients lose interest and get bored while performing similar tasks,^[8]^ which interferes with training performance and thus interferes with clinical outcome. The clinical efficacy of task-oriented training alone is limited to some extent, so some studies have combined rTMS with TOT to further improve overall functional impairment in stroke patients, with improvement of upper limb function being a natural priority. In this paper, we collected clinical trials of rTMS combined with TOT for upper limb function in stroke and conducted Meta-analysis, aiming to further clarify the clinical efficacy of rTMS combined with TOT, guide clinical rehabilitation and improve rehabilitation efficacy.

## 2. Information and methods

### 2.1. Inclusion and exclusion criteria

#### 2.1.1. Study type.

Clinical studies, including 9 randomized controlled studies and 2 non-randomized controlled studies.

#### 2.1.2. Study population.

Stroke patients receiving rTMS and TOT treatment, in which the patients were conscious enough to cooperate with TOT treatment and had upper limb dysfunction.

#### 2.1.3. Interventions.

rTMS combined with TOT treatment.

#### 2.1.4. Outcome indicators.

① Box and block test (BBT).
② Fugl-Meyer assessment (FMA).
③ Modified Barthel index (MBI)
④ Motor evoked potentials (MEPs/duration).

#### 2.1.5. Exclusion criteria.

① Repeatedly reported studies.
② Studies for which the full text could not be read.
③ s Studies with incomplete data information.

### 2.2. Literature search

Computer searches of Pubmed, Cochrane Library, Embase, Web of science, China Knowledge Network, Wanfang, and Vipul databases were conducted from the time of library construction to 2022-10-27. In addition, the references of the included literature were used as a supplement.

### 2.3. Literature screening and data extraction

Two researchers independently extracted information from the included studies using a standardized data extraction form, and any disagreements were discussed thoroughly to reach a consensus. Extracted information included basic study information (original study title, authors, year of publication, original study source), PICO information (demographic characteristics, specific methods of intervention implementation, outcome indicators), methodological components (study design, study duration, etc), and other (whether the original study was funded or not or the source of funding, potential conflicts of interest, key conclusions drawn by the authors).

### 2.4. Quality evaluation of included studies and risk of bias assessment

The methodological quality of the included literature was performed by the 2 authors separately using a modified Jadad scoring scale, including 4 components of random sequence generation, randomization concealment, blinding, and withdrawal and withdrawal, with scores of 1 to 3 considered as low-quality literature and 4 to 7 considered as high-quality literature. Risk of bias was assessed independently by 2 authors according to the Cochrane risk of bias guidelines, and each quality item was classified as low risk, high risk, and unclear risk. Items used to evaluate bias in each trial included random sequence generation, allocation concealment, blinding of subjects and personnel, blinding of outcome assessment, incomplete outcome data, and selective reporting of bias. Included trials were classified as low, high, or moderate quality based on the following criteria: Trials were judged to be low quality if randomization or allocation concealment was evaluated as high risk of bias, and no other items were considered for risk; Trials were judged to be high quality when randomization concealment and allocation concealment were evaluated as low risk of bias and all other items were evaluated as low risk of bias or unclear; and Trials were considered to be of moderate quality if they did not meet the high or low risk criteria.

### 2.5. Statistical analysis

Data analysis was performed using Stata software (https://www.stata.com/).

## 3. Results

### 3.1. Literature search results

A total of 211 papers were retrieved, and 185 papers remained after eliminating duplicates, and 11 papers (5 in English and 6 in Chinese) were finally included after preliminary screening and rescreening. The literature was published from 2013 to 2022, with a total of 520 study cases, including 9 RCTs and 2 non-RCTs, and the literature screening process and results are shown as follows.

### 3.2. Basic characteristics of the included studies

For details, see Table 1.

**Table 1:** Basic characteristics of literature.

Number	Title	Author	Year	State	Experiment design	Design thought	Sample size experimental group/control group	Age	Disease time (mo)	Transcranial magnetic stimulation site	Transcranial magnetic stimulation parameters	Task-oriented training time	Treatment cycle	Primary outcome index	Secondary outcome indicator	Random allocation	Follow-up visit	Conclusion
1	Combining rTMS and task-oriented Training in the rehabilitation of the arm after stroke- a pilot randomized controlled trial	Johanne Higgins	2013	Canada	Single-blind RCT	rTMS combined with task orientation vs Sham combined with task orientation	4/5	74 ± 8	134 ± 125	Motor cortex of the unaffected hemisphere	1200 pulses, 1HZ, 110% intensity	10–15 min	Twice a wk for a mo	1. Box and block test (BBT) 2. Motor evoked potentials (MEPs)	1. Motor function test (WMFT) 2. Motor activity log-14 (MAL-14) 3. Grip strength 4. Pinch strength 5. The stroke impact scale (SIS-16) 6. SIS questionnaire	Yes	Yes	Short- time effective, Long-term invalidity
2	Effect of intensive motor training with repetitive transcranial magnetic stimulation on upper limb motor function in chronic poststroke patients with severe upper limb motor impairment	Yuichi Hirakawa	2018	Japan	Not randomized, not controlled	rTMS combined with intensive exercise training were compared before and after intervention	26	33.1 ± 16.7	33.15 ± 16.43	Mid-sagittal line, unaffected hemisphere	880 pulse, 1HZ, 90% intensity	60 min	Twice a d for 24 d	1. Fugl-Meyer assessment (FMA)	Wolf motor function test (WMFT)	No	No	Effective
3	Effects of high-frequency repetitive transcranial magnetic stimulation combined with task-oriented mirror therapy training on hand rehabilitation of acute stroke patients	Jinhong Kim	2018	Korea	Single-blind RCT	rTMS combined with task-oriented vs rTMS alone	8/12	51 ± 2.98	1.63 ± 0.74	M1, damaged cerebral hemisphere	1500 pulses, 20HZ, 90% intensity	30 min	15 min for 2 wk	1. Motor evoked potentials (MEPs) 2. Box and block test (BBT)	1. Grip strength 2. Pinch strength	Yes	No	Effective
4	Repetitive transcranial magnetic stimulation combined with task-oriented training to improve upper extremity function after stroke	Myoung-Kwon Kim	2014	Korea	Double-blind RCT	rTMS combined with task orientation vs Sham combined with task orientation	16/15	62.40 ± 7.50	3.70 ± 1.25	Motor cortex	10HZ, 80% intensity	30 min	Ten min 5 times a wk for a mo	1. Motor evoked potentials (MEPs) 2. Manual function test(MFT)	null	Yes	No	Effective
5	The effect of 1 Hz repetitive transcranial magnetic stimulation combined with task-oriented training on upper limb function and hemineglect in stroke patients	Hyun Gyu Cha	2017	Korea	Double-blind RCT	rTMS combined with task orientation vs Sham combined with task orientation	12/13	63.92 ± 8.56	2.50 ± 1.51	Motor cortex of the unaffected hemisphere	1200 pulses, 1HZ, 110% intensity	30 min	20 min, 5 days a wk for a mo	1. Fugl-Meyer assessment (FMA) - upper limb 2. Box and block test (BBT) 3. The albert test (AT) 4. Grip strength	Null	Yes	No	Effective
6	Effect of low-frequency repetitive transcranial magnetic stimulation combined with task-guided mirror therapy on upper limb motor function in patients with cerebral infarction	Jin Liu	2018	China	RCT	Conventional rehabilitation control vs task-oriented vs task-oriented combined rTMS	30/30	58.30 ± 14.65	128.73 ± 4.71 (d)	M1 region of the healthy lateral hemisphere	600 pulses, 1HZ, 90% intensity	30 min	15 min 6 times a wk for a mo	1. Motor evoked potential cortical latency (CL) 2. Central motor conduction time (CMCT) 3. Fugl-Meyer assessment-upper extremity (FMA-UE) 4. Modified barthel index (MBI)	Null	Yes	No	Effective
7	Effect of high-frequency repetitive transcranial magnetic stimulation combined with task-oriented training on upper limb motor function rehabilitation in patients with hemiplegia after stroke	Qin Zhao	2022	China	RCT	Simple task orientation vs task Joint rTMS	45/42	62.3 ± 8.3	5.2 ± 3.0	The dorsolateral prefrontal cortex of the affected side	2000 pulses, 10HZ, 80% intensity	40 min	Six wk, 7 d a wk, 20 min	1. Fugl-meyer assessment of upper extremity (FMA- UE) 2. Wolf motor function test (WMFT) 3. Modified Barthel index (MBI)	Null	Yes	Yes	Effective
8	Effect of high-frequency repetitive transcranial magnetic stimulation combined task-oriented training on hand function rehabilitation of stroke patients	Lijuan Deng	2018	China	RCT	Simple rTMS vs simple task-oriented vs task-combined rTMS	38/38	56.01 ± 6.97	61 ± 29.98 (d)	Affected hemisphere	900 pulses, 3HZ, 90% intensity	30 min	30 min 5 times a wk for 6 weeks	1. Fugl-Meyer (finger part, FMA) 2. Modified ashworth score (MAS)	Null	Yes	No	Effective
9	Effect of high-frequency repetitive transcranial magnetic stimulation combined with task-oriented training on limb function in patients with poststroke hemiplegia	Yu Zhang	2020	China	RCT	Simple task orientation vs task Joint rTMS	47/47	61.13 ± 5.26	55.33 ± 7.72 (d)	Motor cortex	1600 pulses, 5HZ, 100% intensity	30 min	30 min 5 times a wk for 6 wk	1. Fugl-Meyer assessment (FMA) - upper limb 2. Modified ashworth score (MAS) 3. Motor evoked potential cortical latency (CL) 4. central motor conduction time (CMCT)	Null	Yes	No	Effective
10	Analysis of the effect of repeated transcranial magnetic stimulation combined with task-oriented training on hemineglect of stroke	Fengzhu Zhao	2020	China	Not RCT	rTMS combined with task-oriented vs rTMS alone	25/25	56.5 ± 1.44	Null	Left posterior parietal cortex	Null	Null	Null	1. Fugl-Meyer (finger part, FMA) 2. Modified Barthel index (MBI) 3. Action research arm test (ARAT)	Null	No	No	Effective
11	Repeated transcranial magnetic stimulation promotes recovery of upper limb motor function after stroke	Hongbin Wang	2017	China	RCT	Simple task orientation vs task joint rTMS	22/20	61.41 ± 8.05	69.27 ± 14.94(Day)	Primary motor cortex M1 on the healthy side	1200 pulses, 1HZ, 90% intensity	40 min	Four wk, 6 times a wk for 20 min	1. Fugl-Meyer assessment (FMA) - upper limb 2. Wolf motor function test (WMFT) 3. Compound muscle action potential (CMAP) 4. Motor evoked potentials (MEPs) 5. Central motor conduction time (CMCT)	Null	Yes	Yes	Effective, but the neurological indicators were not satisfactory

rTMS = repetitive transcranial magnetic stimulation.

### 3.3. Quality evaluation of included studies

**Figure 1.**
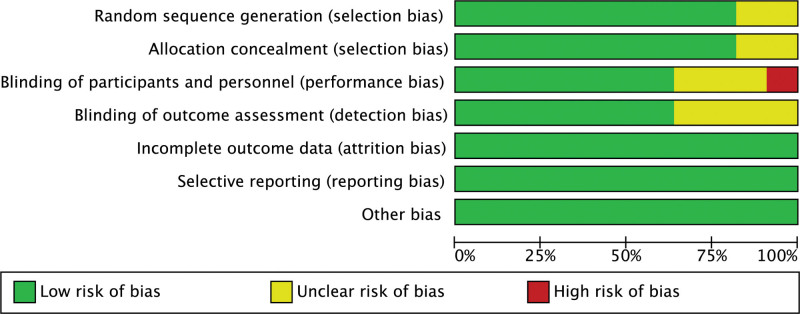
Risk of bias graph.

**Figure 2.**
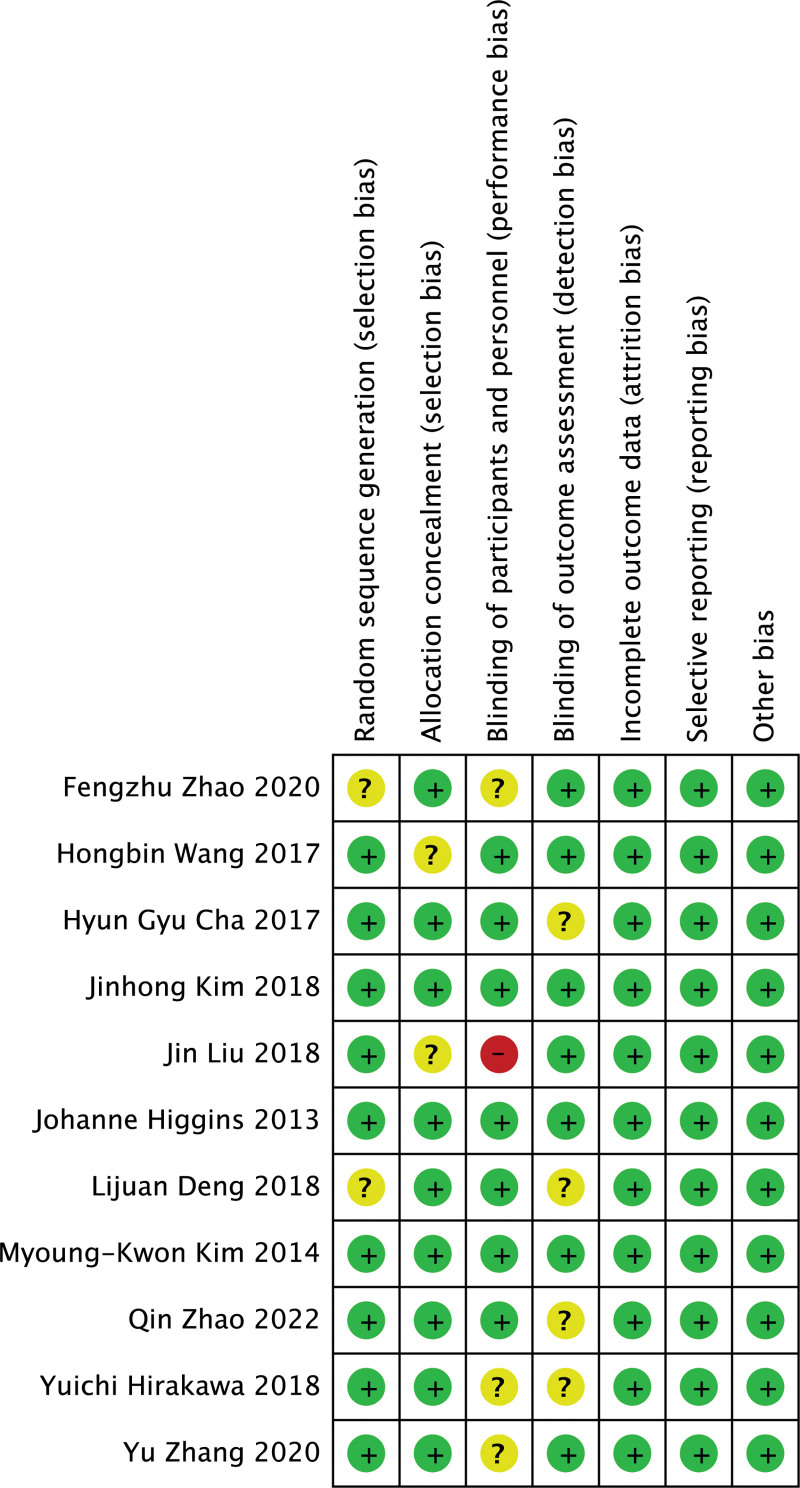
Risk of bias summary.

Nine^[9–17]^ studies mentioned “randomized” and 2^[18,19]^ were non-randomized controlled studies. Seven^[9–14,17]^ did not specify which randomization method was utilized, 1^[15]^ used the random number table method for randomization, 1^[16]^ randomized according to the time of documentation, 1^[19]^ grouped according to patient and family hospital, and 1^[18]^ did not group. Randomized sequences generated 9^[9–14,16–18]^ as low risk and 2^[15,19]^ as unknown risk; allocation concealment was low risk in 9^[9–12,14–16,18,19]^ and unknown risk in 2^[13,17]^; blinding of investigators and subjects was performed in 7^[9–12,14,15,17]^ as low risk, 3^[16,18,19]^ as unknown risk, and 1^[13]^ as high risk; 7^[9–11,13,16,17,19]^ as low risk and 4^[12,14,15,18]^ as unknown risk for blinded evaluation of study outcomes; 11[9,10,11,13,16,17,12] for completeness of outcome data,^[13–19]^ were low risk; selective reporting of study results 11^[9–19]^ were low risk; and other sources 11^[9–19]^ were low risk. Risk of bias plots and risk of bias summary plots are shown below. For details, see Figure 1 and Figure 2.

### 3.4. Meta-analysis results

#### 3.4.1. Effect on stereo block test (box and block test, BBT).

**Figure 3.**
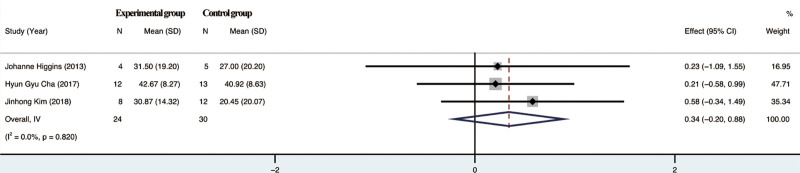
Meta-analysis of BBT. BBT = box and block test.

The 3 included studies reported patients BBT, and the test for heterogeneity suggested *I*²=0%, and the studies showed no heterogeneity, and a random effects model was chosen. the results of the Meta-analysis showed no significant difference between the test group and the control group in terms of improvement of BBT scores by rTMS combined with TOT (*P* = .820), 95% confidence interval (CI) [−0.20,0.88]. The completion of this test requires a certain degree of cognitive ability, but 1 of the 3 studies included did not control cognitive function as a variable, which may greatly affect the test results and cause errors in the test results, resulting in a certain degree of bias in the results of this study. For details, see Figure 3.

#### 3.4.2. Effect on FMA.

**Figure 4.**
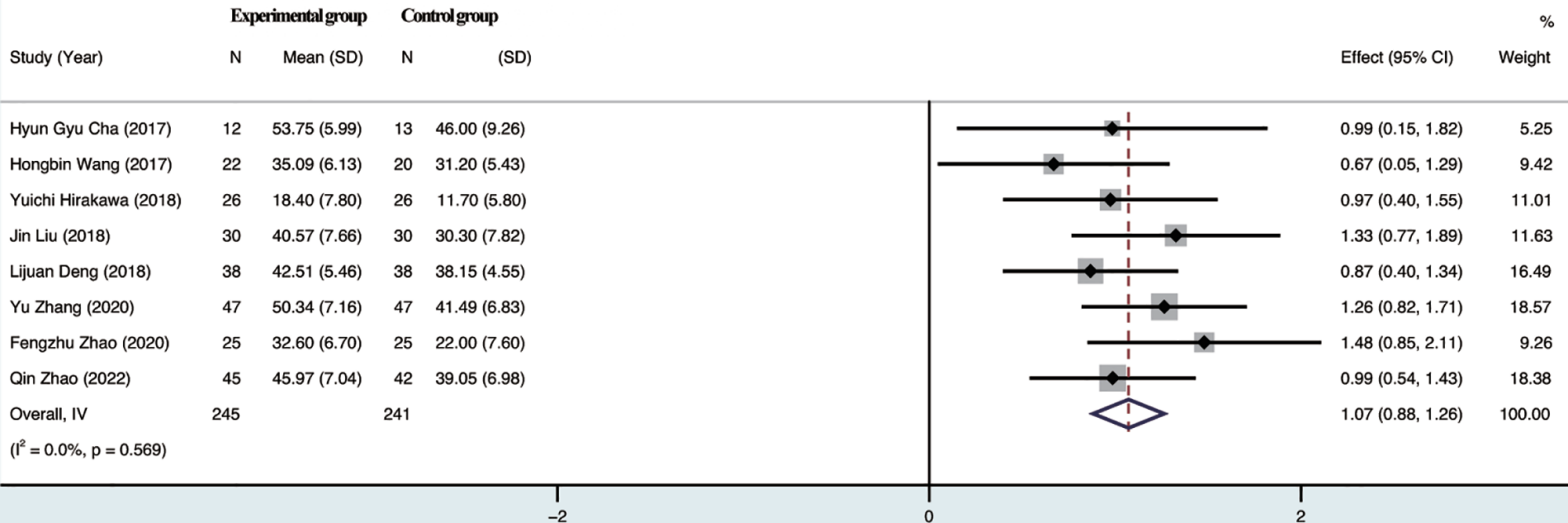
Meta-analysis of FMA. FMA = fugl-meyer assessment.

The 8 included studies reported patients FMA, and the test for heterogeneity suggested *I*²=0%, the studies showed no heterogeneity and a random effects model was chosen. the results of the Meta-analysis showed no significant difference between the trial group and the control group in terms of improvement of FMA scores by rTMS combined with TOT (*P* = .569), 95% CI [0.88,1.26]. For details, see Figure 4.

#### 3.4.3. Effect on MBI.

**Figure 5.**
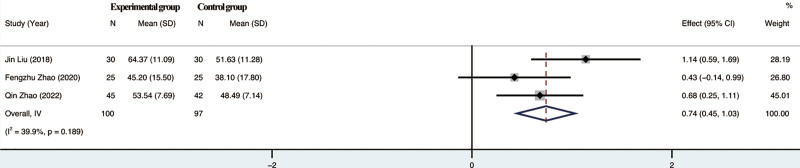
Meta-analysis of MBI. MBI = modified Barthel index.

The 3 included studies reported MBI in patients and the test of heterogeneity suggested *I*²=39.9%, the study showed moderate heterogeneity and a random effects model was chosen. Meta-analysis results showed no significant difference between the trial group and the control group in terms of improvement of MBI scores by rTMS combined with TOT (*P* = .189), 95% CI [0.45,1.03]. For details, see Figure 5.

#### 3.4.4. Effect on MEPs.

**Figure 6.**
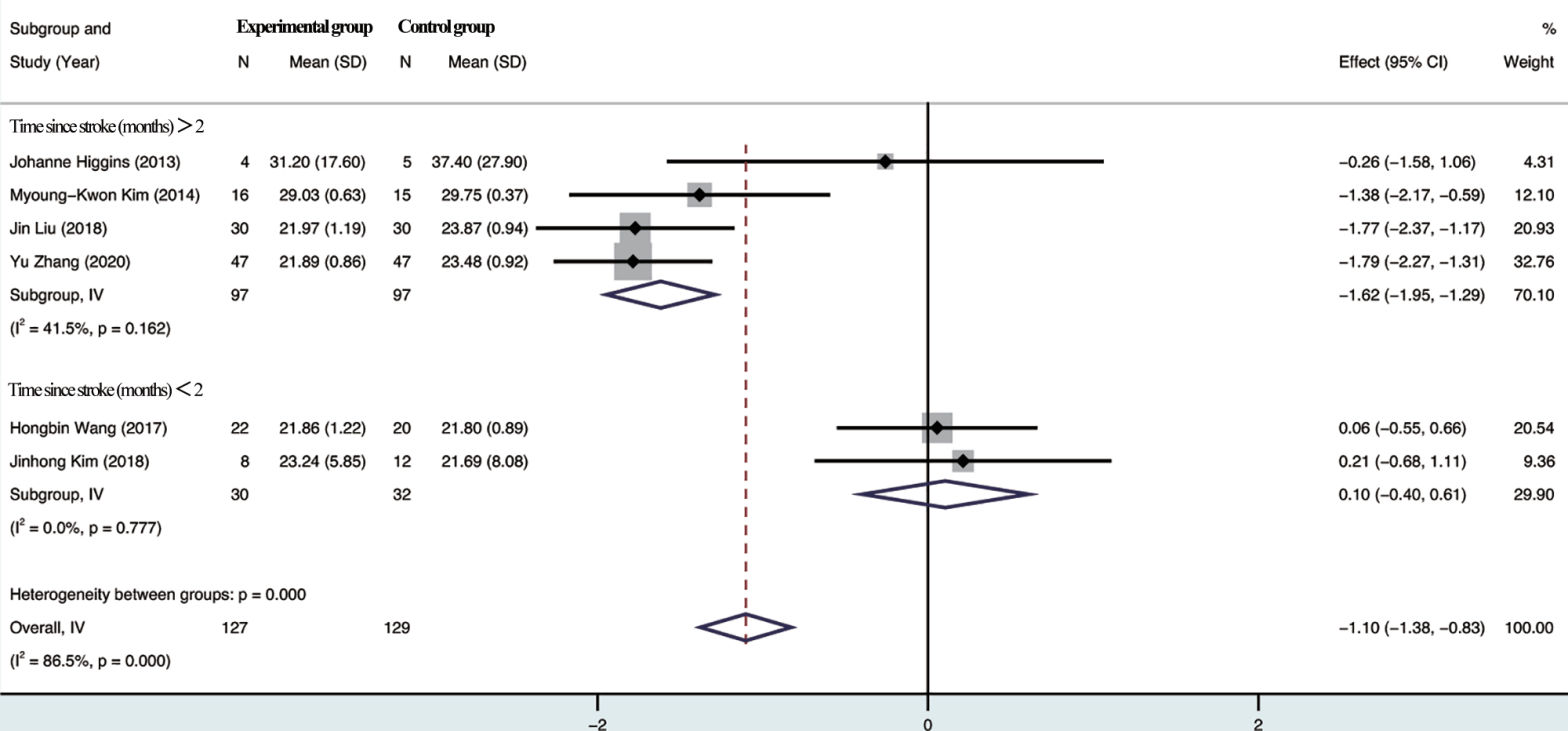
Meta-analysis of MEPs. MEPs = motor evoked potentials.

Six included studies reported MEPs in patients, of which 4 studies with duration of illness longer than 2 months had a heterogeneity test suggesting *I*²=41.5%, showing moderate heterogeneity, and 2 studies with duration of illness <2 months had a heterogeneity test suggesting *I*²=0%, showing no heterogeneity. However, the total heterogeneity of the 6 studies was *I*²=86.5%, and the studies showed high heterogeneity. the results of the Meta-analysis showed that there was a significant difference between the trial group and the control group in terms of improvement of MEPs by rTMS combined with TOT (*P* < .001), 95% CI [−1.38, −0.83]. For details, see Figure 6.

#### 3.4.5. Subgroup analysis of FMA.

**Figure 7.**
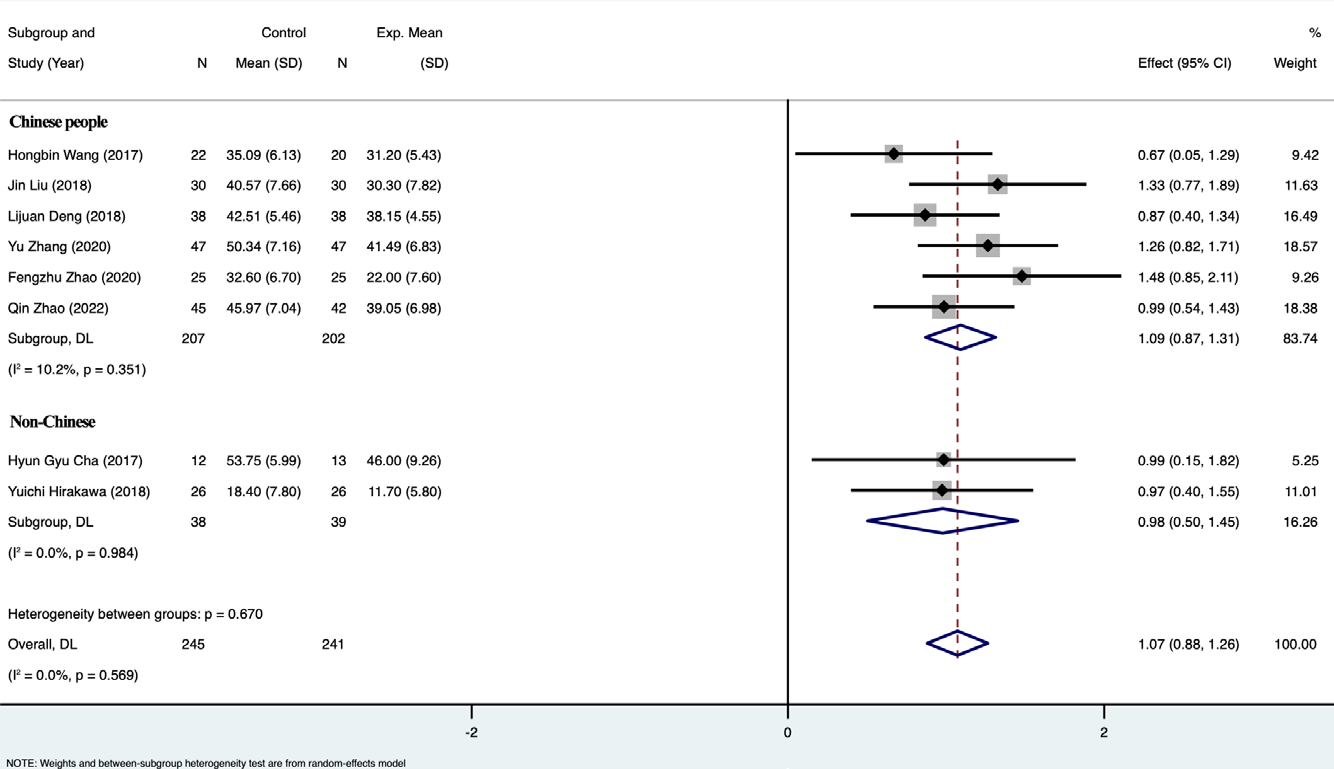
Subgroup analysis of FMA. FMA = fugl-meyer assessment.

**Figure 8.**
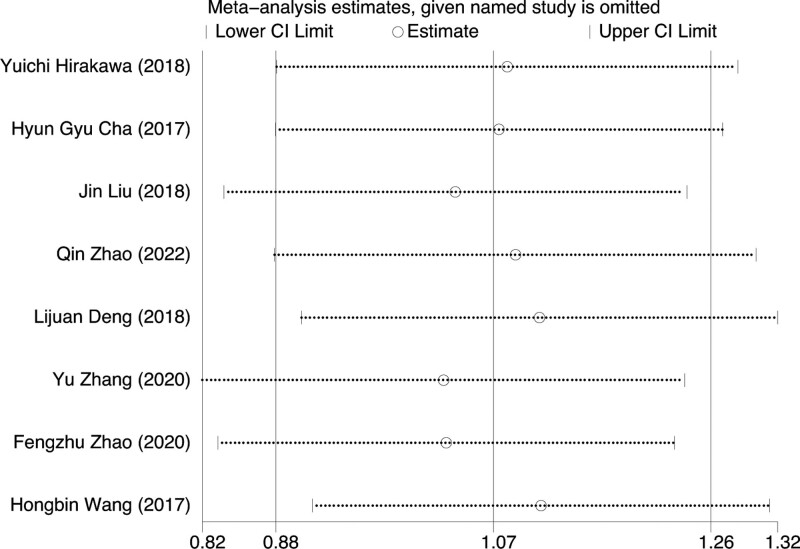
Sensitivity analysis of FMA. FMA = fugl-meyer assessment.

Subgroup analysis of FMA between Chinese and foreigners showed that there was no significant difference among Chinese (*P* = .351), and heterogeneity test suggested that *I*²=10.2%. There was no significant difference among foreigners (*P* = .984), and the heterogeneity test indicated that *I*²=0. There was no significant difference between Chinese and foreigners (*P* = .569), and the heterogeneity test suggested that *I*²=0%. This indicated that there was no significant difference in the improvement of FMA after rTMS and TOT treatment between different races, and there was no racial difference. For details, see Figures 7 and 8.

## 4. Discussion

### 4.1. Significance of the study

Reduced upper limb motor function after stroke severely affects an individual’s ability to perform different tasks of daily living, such as reaching, grasping, and manipulating, which may reduce independence, increase the burden of care, and lead to difficulties in activities of daily living.^[20,21]^ The main rehabilitation therapies for functional rehabilitation of the upper extremity in stroke patients are proprioceptive neuromuscular facilitation techniques, Brunnstorm, Bobath therapy, motor relearning programs, constraint-induced movement therapy, mirror therapy, and TOT.^[22]^ Neuroplasticity-induced cortical reorganization is an important process that mediates the recovery of motor function after stroke.^[23]^ Studies have shown that rTMS improves central neuroplasticity through electromagnetic effects,^[24]^ but it is controversial whether this plasticity can be translated into actual improvement of motor function in practice. Although TOT has been widely used and proven effective among clinics, it remains to be clarified whether the efficacy of combining it with rTMS for upper limb motor function after stroke is more effective than conventional rehabilitation. In this study, 11 studies were included, and the data analysis clarified the efficacy of rTMS combined with TOT on upper limb motor function disorders after stroke, but there was no significant difference between the efficacy of BBT, FMA, and MBI and the efficacy of MEPs in the control group. The combined application of rTMS with other rehabilitation therapies laid the foundation for future application. It affirms the advantages of function-oriented TOT for targeted treatment of functional disorders, promotes the application of TOT in rehabilitation, and improves the rehabilitation efficacy of functional disorders in stroke patients.

### 4.2. Results of the study

This study systematically evaluated the efficacy of repetitive transcranial magnetic stimulation combined with task-oriented training in improving upper extremity dysfunction in stroke patients. The results of the Meta-analysis showed that repetitive transcranial magnetic stimulation combined with task-oriented training improved manual dexterity, upper extremity motor function, activities of daily living and nerve conduction function, which was significantly better than the control group in improving nerve conduction function.

### 4.3. Limitations of the study

The following limitations exist in this study: 2 of the 11 included studies were non-RCTs; and only 4 mentioned and implemented blinding (2 single-blind and 2 double-blind), which cannot exclude the existence of selectivity and implementation bias; Not all of the outcome indicators and control group interventions of the included studies were the same; Only 1 study observed the long-term efficacy. The study was unable to observe differences between chronic and acute effects after treatment; There were several aspects of high heterogeneity high; and Lack of mechanism exploration; The treatment time after stroke will affect the therapeutic effect, but the treatment time included in this study is not uniform, so it will cause a certain degree of error in the results of this study.

### 4.4. Implications for clinical studies

Based on the available Meta-analysis evidence, repetitive transcranial magnetic stimulation combined with task-oriented training may further improve upper limb dysfunction in stroke patients. However, given the high and low quality of the included studies, future relevant studies should improve the methodological quality of the study: clarify the specific random assignment method to achieve concealed grouping and blinded implementation, thus reducing publication bias and improving the level of evidence-based evidence. Strengthen the follow-up so as to clarify the long-term efficacy of the treatment. This study included a low incidence of adverse reactions in the literature, and future safety studies could be focused on.

## 5. Conclusion

In conclusion, repetitive transcranial magnetic stimulation combined with task-oriented training may further improve upper extremity dysfunction in stroke patients, possibly by promoting neuro electrical signal transmission to achieve neurological remodeling. However, due to the sample size and quality of the included studies, the conclusions of this study need to be maintained with caution, and more large, multicenter, double-blind, high quality RCTs are expected to further validate them.

## Author contributions

Data curation: Hujun Wang.

Methodology: Xiaoming Xi.

Resources: Liang Han, Mengmeng Ding, Jinglu Li, Chenye Qiao, Linlin Zhang, Zongjian Liu, Shuyan Qie.

Writing – original draft: Xiaoming Xi.

Writing – review & editing: Hujun Wang.
